# From Seed to Seedling: Constraints Imposed by Warming, UV‐B, and Burial Depth on Alpine Cushion Plant Recruitment

**DOI:** 10.1002/ece3.72814

**Published:** 2026-01-11

**Authors:** Meihong Huang, Pengfei Yang, Jianying Xiang, Mengqiu Niu, Jie Lin, Jianguo Chen

**Affiliations:** ^1^ State Key Laboratory of Plant Diversity and Specialty Crops, Kunming Institute of Botany Chinese Academy of Sciences Kunming China; ^2^ College of Biodiversity Conservation Southwest Forestry University Kunming China; ^3^ School of Ecology and Environment Southwest Forestry University Kunming China; ^4^ University of Chinese Academy of Sciences Beijing China

**Keywords:** climate warming, population dynamics, seed burial depth, seedling emergence, seedling growth, UV‐B radiation

## Abstract

Alpine cushion plants play keystone roles in regulating and sustaining various ecosystem functions, including enhancement and maintenance of community diversity and productivity. Therefore, their population dynamics may exert significant influences on alpine ecosystems. However, the mechanisms driving their population dynamics, particularly during critical early recruitment stages such as seedling emergence and subsequent growth, remain poorly understood. This study investigated how warming temperatures, intense UV‐B radiation, and increased seed burial depth affect the emergence, growth, and mortality of seedlings from 11 cushion plant species in the alpine subnival zones of the Himalaya‐Hengduan Mountains in southwestern China. Results revealed that deep seed burial (exceeding 1 cm) significantly suppressed seedling emergence in cushion species, with substantial loss of seed viability occurring within 3 months for seeds buried at greater depths. Warmed temperatures (> 20°C) accelerated emergence rates and enhanced seedling growth (increased height and leaf number), but also resulted in high seedling mortality rates (ranging from 60% to 100% in most species). In addition, intensified UV‐B radiation consistently impaired seedling recruitment across multiple stages, leading to reduced emergence percentages, stunted growth, and increased mortality rates. These findings suggest that the coarse, mobile substrates characteristic of subnival zones exacerbate natural constraints on seedling emergence in cushion plants. While increased temperatures may promote emergence and growth, they can also result in significant mortality, thereby hindering future population recruitment and expansion. Furthermore, enhanced UV‐B radiation due to ozone layer depletion poses additional threats to cushion populations. Nevertheless, the observed species‐specific responses to these factors indicate that cushion species may exhibit diverse population dynamics under future climate change scenarios. This study provides essential insights into the potential trajectories of alpine cushion populations under climate change, offering valuable implications for the conservation of ecosystems and biodiversity.

## Graphical abstract

This study investigated the effects of warming temperatures, intense UV‐B radiation, and seed burial depth on seedling emergence, growth, and survival of 11 cushion plant species in alpine subnival zones of the Himalaya‐Hengduan Mountains. Deep burial (> 1 cm) severely inhibited emergence in 10 species, with viability loss within 3 months. Warming accelerated emergence and growth but also increased mortality. Intense UV‐B radiation consistently impeded recruitment, reducing emergence, growth, and increasing mortality.

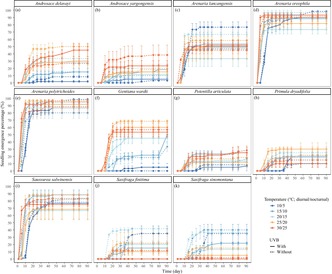

## 1 Introduction

Alpine ecosystems are distinguished by their low temperatures, unstable substrates with poor soil development, intense ultraviolet (UV) radiation, and short growing seasons (Körner 2021). Despite these extreme conditions posing substantial limitations on plant growth and survival, alpine ecosystems harbor remarkable plant diversity (Xu et al. 2014; Sun et al. 2017; Körner 2021). Notably, the presence of numerous cushion plant species, which display a hemispherical or subhemispherical to flat shape due to the dense branching of their shoots and short internodes (Parsons and Gibson 2009), stands out as a pivotal driver of alpine plant diversity that has been globally emphasized (Cavieres et al. 2002, 2014, 2016; Schöb et al. 2012; Chen, Schöb, et al. 2015; Chen, Yang, et al. 2015; Chen et al. 2019; Kikvidze et al. 2015; Gavini et al. 2020). By modulating micro‐environmental conditions, cushion plants create stress‐ameliorated microhabitats, which facilitate the growth and survival of other stress‐intolerant species, enabling them to thrive within or around the cushions' canopies. In turn, this reshapes the structural attributes of plant assemblages and enhances and sustains local and regional plant diversity (Badano and Cavieres 2006; Badano et al. 2006; Schöb et al. 2012, 2013; Chen, Schöb, et al. 2015; Chen, Yang, et al. 2015; Kikvidze et al. 2015; Cavieres et al. 2016). Consequently, the enduring presence of cushion plants in alpine ecosystems plays a crucial role in maintaining alpine plant diversity over the globe. However, cushion plants are notably sensitive to climate change (Cranston et al. 2015; Chen, Chen, et al. 2024; Rai et al. 2024; Jandova et al. 2025), largely due to their status as convergent evolutionary adaptations among phylogenetically unrelated taxa across various cold and/or arid regions worldwide (Aubert et al. 2014; Körner 2021). Thus, their population dynamics may directly impact the community structures and, ultimately, the long‐term preservation of associated non‐cushion plant diversity. Therefore, elucidating the mechanisms driving cushion population dynamics holds significant ecological importance for assessing and predicting the evolution of alpine plant diversity with ongoing climate change.

Global warming has induced certain changes in alpine cushion‐dominant communities. For example, warmed temperatures can exert profound impacts on the physiological processes, growth patterns, and reproductive functions of cushion plants (Cranston et al. 2015; Rai et al. 2024; Jandova et al. 2025), potentially altering even the previously prevalent interspecific facilitative interactions (Cavieres et al. 2024). These changes may further influence the dynamics of cushion species themselves and the communities they dominate. Indeed, a recent research conducted in the Himalaya‐Hengduan Mountain (HHM) region of southwestern China observed that the degeneration of the cushion plant *Arenaria polytrichoides* leads to substantial alterations in the composition of associated species and their relative abundances within these communities. This process may ultimately result in succession toward a sedge‐dominated climax community (Chen, Chen, et al. 2024). The mechanisms driving cushion population dynamics are intricate and likely the culmination of various environmental factors induced by climate change. These factors include direct mortality due to increased temperatures (Zhou et al. 2025), shifts in interspecific competitions (Raath‐Krüger et al. 2023), constraints on seed germination and seedling growth (Chen et al. 2020; Chen, Chen, et al. 2024; Zhou et al. 2025), as well as potential allelopathic effects from surrounding vegetation (Chen, Qian, et al. 2024). Particularly, the early recruitment stages, including seed germination, seedling establishment, and subsequent growth and survival, serve as critical tests for alpine plant populations. These stages determine their persistence or extinction in the face of global change, as they directly influence the likelihood of population recruitment and dynamics of distribution ranges (Gimenez‐Benavides et al. 2008; Donohue et al. 2010; Fernández‐Pascual et al. 2021; Oldfather et al. 2021; Chen, Chen, et al. 2024).

Seedling emergence (or seed germination) and subsequent seedling growth are concurrently influenced by an array of ecological factors, including temperature, moisture, solar radiation, and soil conditions (Benvenuti et al. 2001; Fenner and Thompson 2005; Limón and Peco 2016; Liu et al. 2016; Chen et al. 2020). Among these environmental factors, temperature, moisture, and light might be the most important ones and hence be frequently concerned and widely tested (Fenner and Thompson 2005; Baskin and Baskin 2014; Batlla and Benech‐Arnold 2015; Peng et al. 2018; Chen et al. 2020; Chen, Chen, et al. 2024; Zhou et al. 2025). Besides, UV‐B radiation has also been confirmed to exert significant impacts on seed germination and seedling growth performance, with strong UV‐B radiation generally exerting negative influences on these ecological processes (Ozel et al. 2021). Typically, these factors interact synergistically to affect ecological processes such as seed dormancy, germination, seedling mortality, and the growth and survival of juvenile plants (Muller et al. 2011; Song et al. 2013; Peng et al. 2018), ultimately shaping plant colonization processes. However, alpine plants face a relatively brief growing season (Körner 2021), typically spanning no more than 3 months for most alpine plants. If seeds fail to germinate and emerge promptly when temperature and moisture conditions are optimal, their subsequent growth performance and overwintering capabilities may be compromised, further impacting long‐term population regeneration potential (Chen, Chen, et al. 2024). Therefore, identifying the primary factors influencing these crucial ecological processes is essential for predicting the future population dynamics of cushion plants, particularly in the context of climate warming.

The HHM region stands as a global biodiversity hotspot, known for its remarkable plant diversity, including cushion plants (Myers et al. 2000; Xu et al. 2014; Sun et al. 2017; Zhang et al. 2022). These cushion plants play a significant role in maintaining regional plant diversity (Yang et al. 2010, 2025; Chen, Schöb, et al. 2015; Chen, Yang, et al. 2015; Chen et al. 2019; Yang et al. 2025). However, some cushion plants and their dominant vegetation are already experiencing contractions due to climate change (Huang and Wang 1991; Zhao et al. 2011; Chen, Chen, et al. 2024). Despite efforts to investigate the mechanisms driving cushion population dynamics at early recruitment stages (Chen et al. 2020; Chen, Chen, et al. 2024; Chen, Qian, et al. 2024; Zhou et al. 2025), it is still far from being completely understood how various ecological factors influence the seedling emergence processes and seedling growth dynamics. In addition to temperature, moisture, and light availability, these ecological processes are also influenced by other environmental factors. Notably, UV‐B radiation is getting more intense due to stratospheric ozone depletion under global change, and it is particularly intense in alpine regions where it impacts various ecological processes, including plant reproductive processes and seed germination (Rau and Hofmann 1996; vandeStaaij et al. 1997; Wang et al. 2008; Ballaré et al. 2011; Llorens et al. 2015; Sharma et al. 2017; Jansen et al. 2022). Furthermore, the soils in alpine subnival ecosystems are immature, primarily composed of gravel particles with large interstitial spaces (Zhang et al. 1997). Alpine plants often produce small seeds or dry fruits (Peng et al. 2014) that can easily fall through soil crevices, resulting in deep burial depths. Seed burial depth directly affects seedling emergence timing and rates (Benvenuti et al. 2001; Sun et al. 2010; Limón and Peco 2016; Abbas et al. 2019), potentially due to limitations in light availability for seed germination and soil mechanical resistance for seedling emergence (Limón and Peco 2016; Fernández‐Pascual et al. 2021). To our knowledge, no studies have yet examined the combined effects of temperature, UV‐B radiation, and soil burial depth on the seedling emergence process and subsequent seedling growth performance of cushion plants. This knowledge gap hinders our understanding of cushion plant population dynamics and their driving mechanisms. Therefore, a comprehensive investigation into these factors is crucial for predicting the impacts of future climate change on alpine ecosystems and hence guiding conservation efforts.

In this study, we conducted controlled laboratory experiments to systematically examine the effects of three crucial environmental factors—temperature, UV‐B radiation, and soil burial depth—on the seedling emergence process and subsequent seedling growth performance of 11 alpine cushion plant species native to the HHM region. The primary objectives were to detect the limiting factors influencing population recruitment in diverse cushion plants during early life‐history stages. Uncovering these issues provides important insights for predicting how cushion plant populations of diverse taxa may respond to ongoing global climate change. Given their role as ecosystem engineers in alpine ecosystems (Badano and Cavieres 2006; Badano and Marquet 2008; Kikvidze et al. 2015), understanding the drivers of their population dynamics has crucial implications for the conservation and sustainable management of alpine biodiversity under changing climatic conditions. Specifically, we addressed three fundamental scientific questions: (1) To what extent do temperature fluctuations, UV‐B radiation exposure, and seed burial depth collectively influence key seedling emergence processes, including the time required for initial seedling emergence (T_0_), the time to peak seedling emergence percentage (T_p_), and final seedling emergence percentage (FSEP)? (2) How do these environmental factors affect subsequent seedling growth performance, including seedling height, leaf production, and seedling mortality? (3) Do species‐specific variations exist in responses to particular environmental factors among different cushion plant species, and what are the potential ecological implications of such variations for their population dynamics under future climate change scenarios?

## 2 Materials and Methods

### 2.1 Target Cushion Species

The target cushion species in this study encompasses 11 species belonging to the families Asteraceae, Caryophyllaceae, Gentianaceae, Primulaceae, Rosaceae, and Saxifragaceae (Table 1). In late September 2023, an ample quantity of mature seeds from each species was collected from diverse mountain ranges (seed sources) within the HHM region (Table 1). Specifically, seeds of each cushion species were collected from 20 to 30 cushion individuals and mixed. The habitats of all seed source populations of the selected cushion species are alpine screes, but there may be certain differences in the attributes of the substrates, such as the slope and bed rock (field observation). Seeds or fruits were encapsulated in paper envelopes and conveyed to the laboratory facility, subsequently undergoing a 1‐month period of aerobic dehydration. Subsequently, as necessitated, seeds were meticulously dissected from their pericarps, stored in paper envelopes and maintained at a temperature of 4°C until the initiation of laboratory experimental procedures. Although the prerequisite of cold stratification for the germination of numerous alpine plant species (Fernández‐Pascual et al. 2021), evidence indicates that it seems unnecessary for seeds of cushion *Arenaria* species (Chen et al. 2020; Chen, Chen, et al. 2024; Zhou et al. 2025). We therefore speculated that a nearly 6‐month duration of cold‐dry storage might efficiently alleviate potential seed dormancy in our focal species. Nevertheless, we acknowledge that further research may be needed to investigate whether seeds of different cushion plant species exhibit dormancy and to identify effective methods for breaking it.

**TABLE 1:** Information on the study species.

Family	Genus	Species	Seed size (length × width; mm)	Seed source	Habitat type	Coordinate	Elevation (m)
Asteracea	*Saussurea*	*S. salwinensis*	3.48 × 1.04				
Caryophyllaceae	*Arenaria*	*A. lancangensis*	1.28 × 0.92	Dongdala snow mountain	Alpine screes	29°70′77″ N 98°00′40″ E	5050
Caryophyllaceae	*Arenaria*	*A. polytrichoides*	1.30 × 0.93	Baima snow mountain		28°28′50″ N 99°00′32″ E	4920
Caryophyllaceae	*Arenaria*	*A. oreophila*	1.48 × 1.16				
Gentianaceae	*Gentiana*	*G. wardii*	1.17 × 0.92				
Primulaceae	*Androsace*	*A. delavayi*	1.55 × 1.01				
Primulaceae	*Androsace*	*A. yargongensis*	1.65 × 1.27	Bayankela snow mountain		34°12′56″ N 97°65′98″ E	4810
Primulaceae	*Primula*	*P. dryadifolia*	1.56 × 1.25	Baima snow mountain		28°28′50″ N 99°00′32″ E	4920
Rosaceae	*Potentilla*	*P. articulata*	2.00 × 1.05	Zheduoshan snow mountain		30°08′48″ N 101°80′68″ E	4500
Saxifragaceae	*Saxifraga*	*S. finitima*	0.58 × 0.31	Baima snow mountain		28°28′50″ N 99°00′32″ E	4920
Saxifragaceae	*Saxifraga*	*S. sinomontana*	0.78 × 0.47				

*Note:* Seed size was averaged from measurements of 15 randomly selected seeds of each species using an anatomical lens.

### 2.2 Laboratory Experiments

Laboratory experiments were conducted utilizing five incubators, corresponding to five temperature gradients (see following). The seedling cultivation medium was composed of soils sourced from Pujin pasture on the Baima Snow Mountain—the native habitat of the eight studied cushion plant species (Table 1)—mixed with perlite in a 5:1 volume ratio, with particles approximately 0.5 cm in diameter. Prior to use, the substrate was thoroughly sterilized by spraying with an 80% carbendazim solution to eliminate potential contaminants.

Field measurements (unpublished data) indicated that during the peak growing season (July to September) for local plants in the Baima snow mountains in 2020, the soil temperature at a depth of 2 cm at the study site ranged from a minimum of 1.8°C to a maximum of 13.5°C, with an average temperature of 6.8°C. However, occasional soil temperature could reach above 25°C in sunny days in a nearby community (Chen, Yang, et al. 2015). While previous research has suggested that a temperature regime of 15°C/10°C (diurnal/nocturnal) is optimal for seed germination and seedling establishment in cushion species *A. polytrichoides* and *A. oreophila* (Chen, Chen, et al. 2024; Zhou et al. 2024), the thermal requirements for other cushion species remain unexplored. Therefore, we designed the incubators with the following temperature settings: 10°C/5°C (diurnal/nocturnal), 15°C/10°C, 20°C/15°C, 25°C/20°C, and 30°C/25°C, respectively. In addition, each incubator was equipped with a 12‐h light cycle (diurnal; 7000 lx) and a 12‐h dark cycle (nocturnal) to mimic natural conditions.

We conducted seed germination and seedling cultivation experiments using plastic trays. Each tray contained six individual cells (5 cm × 5 cm × 8 cm per cell). Every cell was filled with the previously described soil mixture (i.e., seedling cultivation medium), and 20 mature seeds were sown in each cell. To assess the influence of seed burial depth on seedling emergence percentage and subsequent growth, we established three distinct burial depth levels: 0 cm (seeds placed directly on the soil surface), 1 cm (seeds sown 1 cm below the soil surface), and 2 cm (seeds sown 2 cm below the soil surface).

Furthermore, to evaluate the potential effects of UV‐B radiation on seedling emergence and performance, we designated an additional treatment group for each species at each temperature and burial depth level. Given the high frequency of rainy and foggy days during the growing season in the HHM region, which implies that the duration of full UV‐B exposure did not align with the total daytime length, a weekly UV‐B irradiation regimen was implemented for this particular group. The regimen consisted of 36 h of exposure at an intensity of 170 μW/cm^2^, administered over three consecutive nocturnal periods in the absence of light. Notably, the field‐measured UV‐B intensity at the Pujin site during the hours of 13:00 to 16:00 on July 20, 2023, ranged from 150 to 180 μW/cm^2^. To guarantee that the control group (without UV‐B treatment) remained unexposed to UV‐B radiation, we constructed protective covers using double‐layer tin foil that snugly encapsulated the seedling pots (i.e., trays) during the periods of UV‐B treatment. Each treatment group, including all relevant combinations of burial depth, temperature, and UV‐B exposure, was replicated three times (i.e., tray cells).

The seedling trays, each containing the sown seeds, were randomly assigned to the five incubators. The trays were regularly watered with tap water throughout the experiment to exclude potential moisture constraints on seedling emergence or growth. Every 5 days, we checked the trays to meticulously record the number of emerged seedlings and to monitor subsequent seedling performance until the end of the experiment. However, given that the seeds achieved their respective peak seedling emergence percentages within 45 days under almost all experimental conditions, with no further emergence observed during the subsequent 10‐day period from Day 45 to Day 55, we ceased regular 5‐day interval measurements from Day 45. Subsequently, three additional irregular measurements (Day 57, 72, and 79, respectively) were conducted prior to the end of the experiment. Throughout this period, consistent watering and UV‐B treatments were maintained until the end of the experiment on the 90th day, when the final measurements were performed. Specific parameters for assessing seedling growth performance were documented as follows: number of surviving seedlings (the total count of viable seedlings in each tray hole); seedling height (for each tray hole containing more than five seedlings, we selected the five tallest individuals and measured their height from the shoot base to the leaf apex); number of leaves (similarly, for the same five selected seedlings per tray hole, the number of leaves was recorded). This process was consistently followed for a period of 3 months, beginning on April 18, 2024, and ending on July 16, 2024.

Due to the absence of seedling emergence for nearly all the target cushion species, with the exception of *Saussurea salwinensis*, when seeds were buried at depths of one and two centimeters below the soil surface throughout the experiments, we conducted supplementary tests to determine the viability of the seeds at these burial depths. To achieve this, we retrieved the soil from the treatment groups with seeds buried at 1 and 2 cm depths, combined them thoroughly, and evenly spread the mixture onto larger plastic trays with a soil layer approximately 1 cm thick. These trays were then placed back into the incubators. Given the results in this study (see Section 3) suggested that higher temperatures can stimulate seedling emergence, we adjusted the incubator temperature to 25°C/20°C for this supplementary experiment. We ensured that the soil maintained the necessary moisture conditions for seedling emergence by regularly spraying it with an appropriate amount of tap water. Daily checks were conducted to observe any emerging seedlings. The supplementary experiment lasted for a duration of 35 days.

### 2.3 Data Analyses

Given that seedling emergence was observed only for *S. salwinensis* among the target cushion species when seeds were buried at depths of one and two centimeters below the soil surface until the end of the experiments, we excluded the seed burial depth treatment from subsequent data analyses. Consequently, our analyses focused solely on the dataset corresponding to the 0‐cm burial depth treatment. To evaluate the impacts of temperature, UV‐B radiation, and their potential interaction on seedling emergence and subsequent growth performance, we selected six parameters: (i) time to initiation of seedling emergence (T_0_; the duration until the first seedling emerges); (ii) time to maximum seedling emergence percentage (T_p_; the period required to reach the peak seedling emergence rate); (iii) final seedling emergence percentage (FSEP; calculated as the number of emerged seedlings divided by the total number of sown seeds, multiplied by 100%); (iv) seedling height (the measured height of seedlings at the end of the experiment); (v) seedling leaf number (the count of leaves per seedling at the end of experiment); (vi) final seedling mortality rate (FSMR; determined by dividing the number of dead seedlings by the total number of initially emerged seedlings, multiplied by 100%). Since the T_0_, T_p_, FSEP, and FSMR were non‐normal and non‐continuous variables, we used generalized linear mixed models (GLMMs) to evaluate the effects of temperature, UV‐B radiation, plant species, and their potential interactions on these response variables. Poisson link functions were applied for modeling T_0_ and T_p_, while binomial link functions were used for FSEP and FSMR. Model diagnostics were performed by examining the distributions of the residuals to ensure appropriate fit. Additionally, for the analysis of seedling growth parameters (seedling height and leaf number), linear mixed‐effects models were employed. Temperature, UV‐B radiation, species, and their interactions were treated as fixed effects, with experimental replicates included as a random effect. To meet the assumptions of normality and homoscedasticity, the growth parameters were square‐root transformed prior to analysis. Firstly, we formulated complete models, with each of the six parameters serving as response variables. Subsequently, we analyzed separate datasets for each species using similar models.

All statistical analyses described were performed using R version 4.4.3 (R Core Team 2023). Specifically, generalized linear mixed models were conducted utilizing the “glmer” function within the “lme4” package (Bates et al. 2015), and linear mixed‐effect models were conducted utilizing the “lme()” function within the “nlme” package (Pinheiro et al. 2023). For post hoc pairwise comparisons, we leveraged the “emmeans” package in R (Lenth 2024), applying Tukey's honestly significant difference (HSD) method for multiple comparison adjustments. Given the experimental design's limited sample size (with three or fewer replicates per group), we chose to use unadjusted *p*‐values to preserve statistical power. We acknowledge that this decision might elevate Type I error rates but consider it reasonable given the exploratory nature of the study and the biological context, where false negatives could have more significant consequences than false positives. All comparisons were conducted at the nominal significance level (*α* = 0.05), ensuring family‐wise error rate control within each set of treatment contrasts. Additionally, we utilized the “ggplot2” package (Wickham 2016) for creating all the figures.

## 3 Result

### 3.1 Seedling Emergence

With the exception of *S. salwinensis*, no seedling emergence was observed for any of the target cushion species when their seeds were buried at depths of 1 or 2 cm. Specifically, for *S. salwinensis*, a few seedlings emerged at all burial depths. Further supplemental viability tests revealed limited emergence for only five species: *S. salwinensis* with 30 seedlings, *G. wardii* with 4 seedlings, *A. yargongensis* with 3 seedlings, *A. delavayi* with 9 seedlings, and *P. articulata* with 1 seedling. No new seedling emergence was observed for any other species.

**FIGURE 1:**
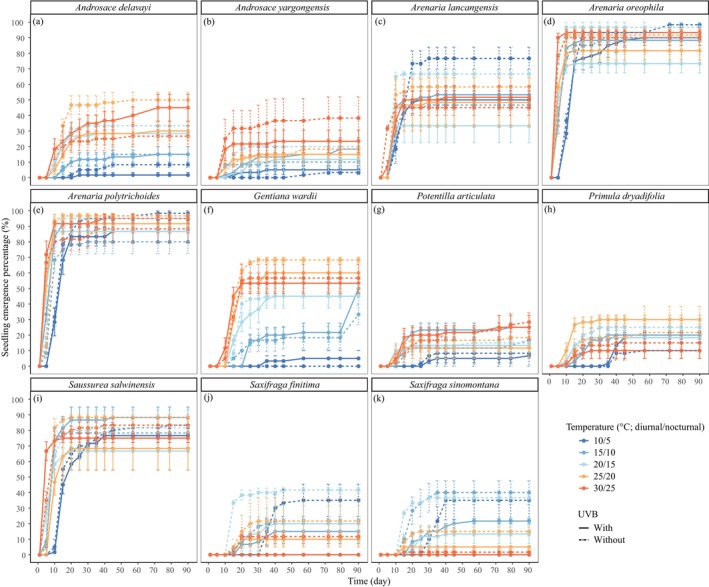
Seedling emergence percentage along with cultivating time under indicated temperature and UV‐B radiation conditions. (a) *Androsace delavayi*; (b) *Androsace yargongensis*; (c) *Arenaria lancangensis*; (d) *Arenaria oreophila*; (e) *Arenaria polytrichoides*; (f) *Gentiana wardii*; (g) *Potentilla articulata*; (h) *Primula dryadifolia*; (i) *Saussurea salwinensis*; (j) *Saxifraga finitima*; (k) *Saxifraga sinomontana*.

The results show significant variation in seedling emergence dynamics and subsequent growth performance among the target cushion species when seeds were placed at a 0‐cm soil burial depth (*p* < 0.001; Table 2; Table S1). Temperature significantly influenced both the time to initial seedling emergence (T_0_) and the time to reach the maximum seedling emergence percentage (T_p_) (*p* < 0.01). Conversely, UV‐B radiation and its interaction with temperature had negligible effects on most species (Table 2A,B; Table S,1A,B). Interspecific differences in T_0_ and T_p_ were highly significant (*p* < 0.001; Table 2A,B). Generally, warmer temperatures accelerated seedling emergence, whereas colder temperatures delayed it (Figure 1; Figure S1). However, a contrasting pattern emerged for the two *Saxifraga* species and *A. lancangensis*, where higher temperatures appeared to hinder seedling emergence, particularly when UV‐B radiation was present (Figure 1j,k).

**TABLE 2:** Results of generalized linear mixed models (for T_0_, T_p_, FSEP, FSMR) and linear mixed‐effects models (for seedling height and leaf number) testing the effects of temperature, UV‐B radiation, species, and their interactions.

Factor	Df	Chisq	Pr(>chisq)
*(A) Time to initial germination*			
(Intercept)	1	1867.209	< 0.001
Temperature	4	220.115	< 0.001
UV‐B	1	0.075	0.784
Species	10	666.201	< 0.001
Temperature × UV‐B	4	4.623	0.328
*(B) Time to maximum germination*			
(Intercept)	1	5577.810	< 0.001
Temperature	4	240.227	< 0.001
UV‐B	1	0.136	0.712
Species	10	463.288	< 0.001
Temperature × UV‐B	4	15.063	0.005
*(C) Final seedling emergence percentage*			
(Intercept)	1	211.851	< 0.001
Temperature	4	73.887	< 0.001
UV‐B	1	43.294	< 0.001
Species	10	1683.247	< 0.001
Temperature × UV‐B	4	49.391	< 0.001

Factor	numDF	denDF	*F*‐value	*p*
*(D) Seedling height*				
(Intercept)	1	275	5344.57	< 0.001
Temperature	4	275	4.19	0.003
UV‐B	1	275	52.93	< 0.001
Species	10	275	44.68	< 0.001
Temperature × UV‐B	4	275	3.30	0.012
*(E) Seedling leaf number*				
(Intercept)	1	275	4358.07	< 0.001
Temperature	4	275	11.42	< 0.001
UV‐B	1	275	34.22	< 0.001
Species	10	275	6.01	< 0.001
Temperature × UV‐B	4	275	3.02	0.018

Factor	Df	Chisq	Pr(>chisq)
*(F) Final seedling mortality*			
(Intercept)	1	14.915	< 0.001
Temperature	4	81.629	< 0.001
UV‐B	1	16.335	< 0.001
Species	10	481.132	< 0.001
Temperature × UV‐B	4	10.018	0.040

**FIGURE 2:**
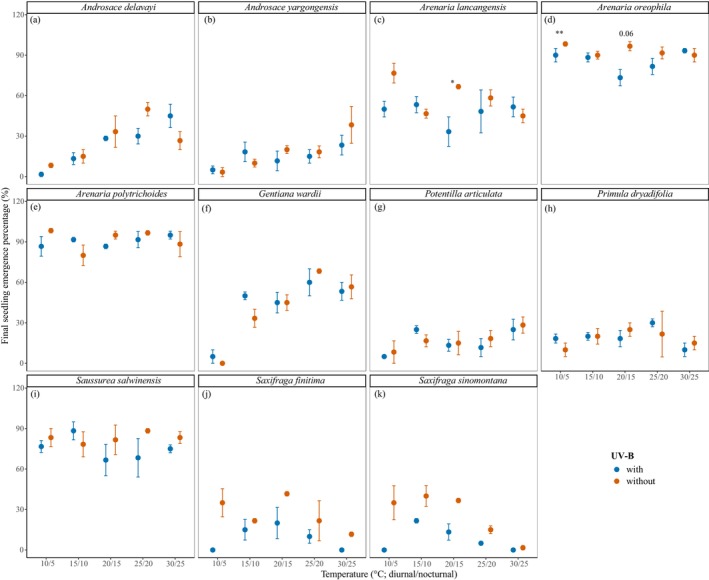
Final seedling emergence percentage of seeds across cushion plant species under different temperature conditions (*x*‐axis) and UV‐B treatments (gold: with UV‐B; blue: without UV‐B). (a) *Androsace delavayi*; (b) *Androsace yargongensis*; (c) *Arenaria lancangensis*; (d) *Arenaria oreophila*; (e) *Arenaria polytrichoides*; (f) *Gentiana wardii*; (g) *Potentilla articulata*; (h) *Primula dryadifolia*; (i) *Saussurea salwinensis*; (j) *Saxifraga finitima*; (k) *Saxifraga sinomontana*. Asterisks indicate significant differences between UV‐B treatments at the same temperature (*p < 0.05; **p < 0.01).

Both temperature and UV‐B radiation, as well as their interaction, have significant impacts on final seedling emergence percentages (*p* < 0.001; Table 2C; Figure 2). Generally, higher temperatures and the absence of UV‐B radiation led to greater seedling emergence, though there was notable interspecific variation (Figure 2; Table S1C). The effect of temperature was especially evident in four species: *A. delavayi* (Figure 2a), *A. oreophila* (Figure 2d), *G. wardii* (Figure 2f), and *P. articulata* (Figure 2g), all of which showed significantly enhanced seedling emergence under warmer conditions (*p* < 0.05; Table S1C). Conversely, seven species—*A. yargongensis* (Figure 2b), *A. lancangensis* (Figure 2c), *A. polytrichoides* (Figure 2e), *P. dryadifolia* (Figure 2h), *S. salwinensis* (Figure 2i), *S. finitima* (Figure 2j), and *S. sinomontana* (Figure 2k)—maintained consistent emergence percentages across all temperature treatments (*p* > 0.05; Table S1C). Significant effects of UV‐B radiation and its interaction with temperature (*p* < 0.05) were observed only in three species: *A. lancangensis*, *A. oreophila* , and *A. polytrichoides*. The remaining species did not exhibit any significant response to UV‐B treatments (*p* > 0.05; Table S1C; Figure 2).

### 3.2 Seedling Growth

**FIGURE 3:**
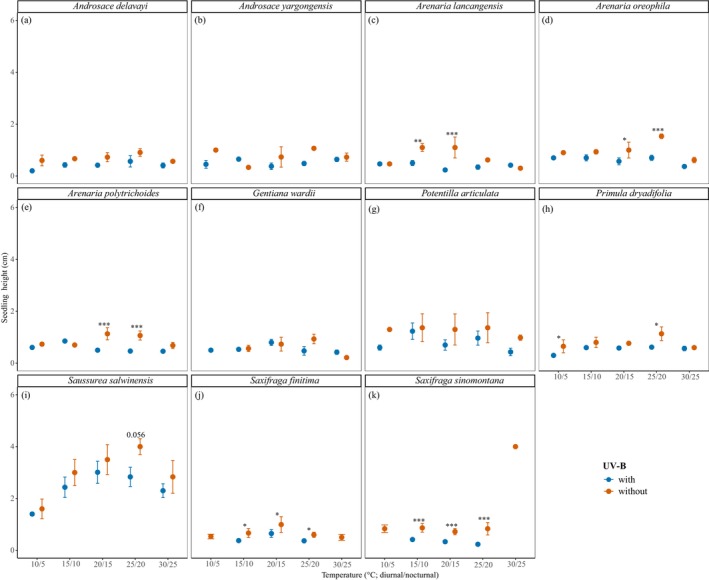
Seedling height under different temperature and UV‐B treatments at the end of experiment. For seedlings that died before the end of experiment, the last recorded height measurements are shown. (a) *Androsace delavayi*; (b) *Androsace yargongensis*; (c) *Arenaria lancangensis*; (d) *Arenaria oreophila*; (e) *Arenaria polytrichoides*; (f) *Gentiana wardii*; (g) *Potentilla articulata*; (h) *Primula dryadifolia*; (i) *Saussurea salwinensis*; (j) *Saxifraga finitima*; (k) *Saxifraga sinomontana*. Asterisks denote significant differences between UV‐B treatments at the same temperature (*p < 0.05; **p < 0.01; ***p < 0.001).

**FIGURE 4:**
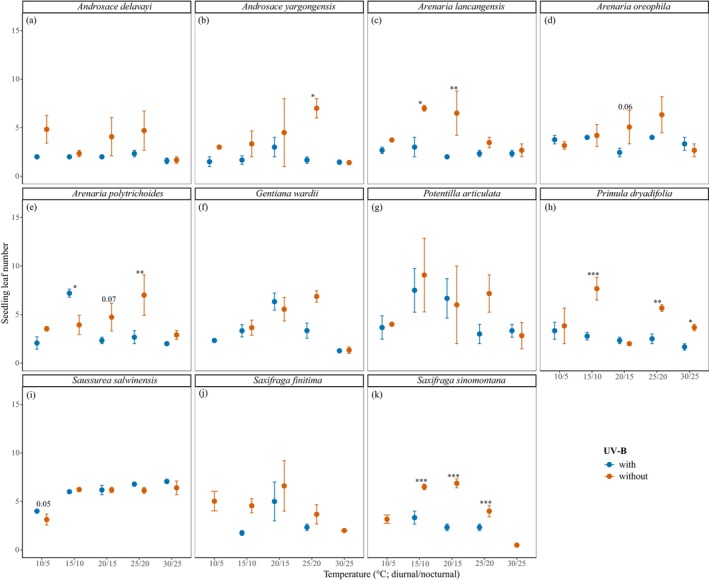
Leaf number of seedlings grown under different temperature and UV‐B treatments at the end of experiment. For seedlings that died prior to the end, the last recorded leaf counts are shown. (a) *Androsace delavayi*; (b) *Androsace yargongensis*; (c) *Arenaria lancangensis*; (d) *Arenaria oreophila*; (e) *Arenaria polytrichoides*; (f) *Gentiana wardii*; (g) *Potentilla articulata*; (h) *Primula dryadifolia*; (i) *Saussurea salwinensis*; (j) *Saxifraga finitima*; (k) *Saxifraga sinomontana*. Asterisks indicate significant differences between UV‐B treatments at each temperature (*p < 0.05; **p < 0.01; ***p < 0.001).

Seedling growth traits, specifically height and leaf number, displayed significant but species‐specific responses to temperature, UV‐B radiation, and their interaction (*p* < 0.05; Table 2D,E; Table S1; Figures 3 and 4). Significant enhancements in height (*p* < 0.05) were observed in *A. lancangensis*, *A. oreophila* , *P. dryadifolia*, *S. salwinensis*, and *S. sinomontana* (Table S1; Figure 3). UV‐B radiation generally suppressed height growth (*p* < 0.05) in most species, with exceptions being *A. yargongensis*, *G. wardii* , and *P. articulata* , which did not show any significant response (*p* > 0.05). In terms of leaf number development, warmed temperatures significantly increased leaf counts (*p* < 0.05) in *A. polytrichoides*, *G. wardii* , *P. dryadifolia*, *S. salwinensis*, and both *Saxifraga* species ( *S. finitima* and *S. sinomontana*). UV‐B exposure had a negative impact on leaf production (*p* < 0.05) in *A. yargongensis*, *A. lancangensis*, *A. polytrichoides*, *P. dryadifolia*, and *S. sinomontana* (Table S1; Figure 4). The remaining species maintained consistent leaf numbers across both temperature and UV‐B treatments (*p* > 0.05; Table S1).

### 3.3 Seedling Mortality

**FIGURE 5:**
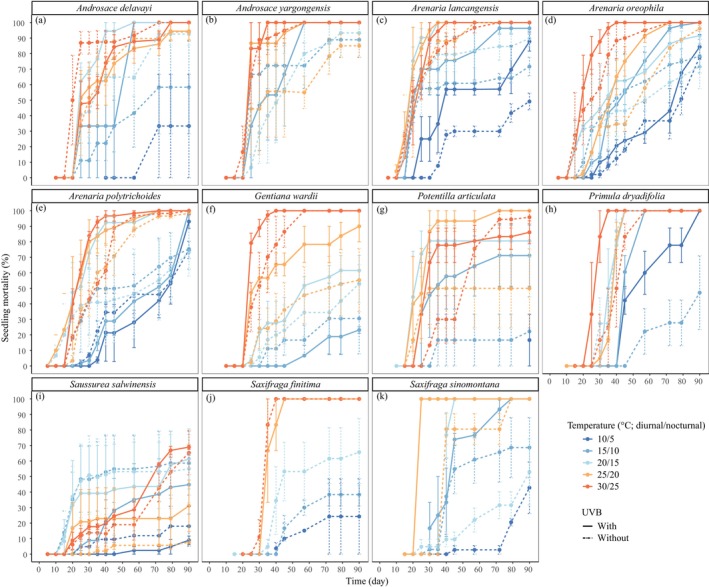
Seedling mortality rates under indicated temperature and UV‐B radiation conditions during cultivation. (a) *Androsace delavayi*; (b) *Androsace yargongensis*; (c) *Arenaria lancangensis*; (d) *Arenaria oreophila*; (e) *Arenaria polytrichoides*; (f) *Gentiana wardii*; (g) *Potentilla articulata*; (h) *Primula dryadifolia*; (i) *Saussurea salwinensis*; (j) *Saxifraga finitima*; (k) *Saxifraga sinomontana*.

Increased temperatures generally accelerated seedling mortality processes (Figure 5) and significantly increased final mortality percentages for the majority of cushion species studied (*p* < 0.05; Table 2F; Figure S2). Due to insufficient data for particular species, formal statistical tests could not be performed (Table S1F); however, a warming‐induced mortality effect was visually apparent in most species. Only *S. salwinensis* exhibited relatively low mortality under warmer conditions (> 20°C) (Figure S2). These species exhibited significant additional mortality increases when exposed to UV‐B radiation (Figure 5; Figure S2).

## 4 Discussion

Cushion plants serve as keystone ecosystem engineers in alpine ecosystems, playing pivotal roles in modulating and sustaining ecological processes through both direct and indirect pathways (Badano and Cavieres 2006; Badano and Marquet 2008; Schöb et al. 2012, 2013; Chen, Schöb, et al. 2015; Chen, Yang, et al. 2015; Chen et al. 2019; Kikvidze et al. 2015). Given their importance, understanding how complex environmental factors collectively determine the population dynamics of these plants is crucial for predicting future trajectories of alpine ecosystems. This study demonstrates that warmed temperatures and intense UV‐B radiation generally impose significant constraints on seedling emergence and subsequent growth across multiple cushion plant species. Notably, we identified soil burial depth as a critical limiting factor, with deeper seed placement severely restricting seedling emergence. However, interspecific variation in responses to these environmental factors is evident.

### 4.1 Seedling Emergence

The seed size of the target cushion species ranges from 0.58 × 0.31 mm ( *S. finitima* ) to 3.48 × 1.04 mm (*S. salwinensis*) in length and width (Table 1). These relatively small seeds may be highly vulnerable to burial within deep soil layers in alpine environments, due to the loose and poorly developed gravel layers with relatively large pore sizes and hence interstitial spaces (Zhang et al. 1997). Our results, although not quantitatively analyzed due to the complete absence of seedling emergence from deeply buried seeds for most cushion species, suggest that burial depths exceeding one centimeter strongly inhibit seedling emergence in most cushion species. This observation aligns with previous studies demonstrating negative effects of deep seed burial on germination success (Benvenuti et al. 2001; Abbas et al. 2019; Tao et al. 2022; Sousa‐Ortega et al. 2023). This highlights the sensitivity of these species to burial depth and underscores the importance of appropriate seed burial depth for successful seedling emergence and establishment of these alpine cushion plants. Several mechanisms may explain this phenomenon. Firstly, light inhibition in deep soil prevents the initiation of seed germination. Light is a critical environmental cue for breaking seed dormancy and initiating germination for most alpine plants (Fernández‐Pascual et al. 2021). However, light penetration is limited to the top few millimeters of soil (Tester and Morris 1987). Consequently, the absence of light at greater depths can prevent or delay germination, thus contributing to the formation of a persistent soil seed bank (Peng et al. 2021). Although we cannot confirm whether our target species possess strong dormancy mechanisms, all species demonstrated emergence capability when surface‐sown (0‐cm depth) under appropriate temperatures (Figure 1). Therefore, we speculate that the seedling emergence of most alpine cushion plants is, to some extent, light cue‐dependent. Secondly, the mechanical resistance of the soil may prevent seedlings buried in deep soil from breaking through the surface. This is highly plausible because the relatively small seeds of most cushion plants (Table 1) are likely to produce small and physiologically weak seedlings with limited resource reserves, thereby reducing their capacity to survive long enough to reach the soil surface (Bond et al. 1999). The observation that few *S. salwinensis* seedlings emerged from deep burial depth supports this hypothesis. Notably, this species possesses the largest seeds among the studied cushion plants (Table 1). It is well established that larger seeds generally produce more robust seedlings, which are better equipped to overcome environmental stresses (Fenner and Thompson 2005). Collectively, we argue that both photoinhibition and mechanical resistance likely contribute to the emergence failure of deeply buried cushion seeds. These findings have important ecological implications: substantial seed loss to deeper soil layers may severely compromise future population recruitment of most alpine cushion plants. This occurs through both the immediate inhibition of germination and the progressive loss of seed viability during prolonged burial. Our supplementary tests support this, indicating that deeply buried seeds are likely to lose viability if they fail to germinate or if emerging seedlings cannot reach the soil surface.

Alpine ecosystems are characterized by short growing seasons (Körner 2021), necessitating precise seedling emergence timing for survival and establishment (Chambers et al. 1990; Schütz 2002; Forbis 2003; Chen, Chen, et al. 2024). Our study demonstrates that warmed temperatures generally accelerated the seedling emergence process in most cushion species when seeds were surface‐sown, promoting both earlier germination onset and faster reaching of maximum germination percentages (Figure 1). However, species‐specific responses were evident in final seedling emergence percentages. While certain species exhibited consistently high emergence percentages irrespective of temperature (Table S1; Figure 1; Figure S1), warming significantly increased seedling emergence in *A. delavayi*, *A. oreophila* , *G. wardii* , and *P. articulata* . In contrast, UV‐B radiation, though not affecting emergence timing, exerted predominantly negative effects (significant or marginal) on final seedling emergence across most species (Figure 1; Figure S1). These findings corroborate previous reports that warming facilitates germination in alpine plants by alleviating thermal constraints (Fernández‐Pascual et al. 2021; Notarnicola et al. 2023; Chen, Chen, et al. 2024; Zhou et al. 2025). For example, seed germination of *A. polytrichoides* and *A. oreophila* is accelerated by warming temperatures under favorable hydration conditions (Chen, Chen, et al. 2024; Zhou et al. 2024). A global meta‐analysis further highlights the pivotal role of warm, fluctuating temperatures and light in triggering alpine seed germination (Fernández‐Pascual et al. 2021). Notably, our data reveal an exception: *Saxifraga* species exhibited suppressed seedling emergence under warming and UV‐B exposure (Figure 1j,k), suggesting taxon‐specific sensitivities. The suppressed emergence of *Saxifraga* seedlings under warming temperatures may indicate potential challenges in the initial critical stage of population recruitment in the context of ongoing climate warming. However, the physiological mechanisms governing dormancy breakage and germination initiation remain complex (Baskin and Baskin 2014) and are not fully resolved in this study, warranting future investigation. While we focus on emergence responses rather than underlying dormancy mechanisms, our results highlight how environmental shifts may alter recruitment dynamics in cushion plant populations. We propose that climate warming is likely to promote seedling emergence in most alpine cushion species, with the exception of certain taxa—such as the *Saxifraga* species observed in this study. However, stratospheric ozone depletion and the associated increase in UV‐B radiation (Montzka et al. 1999) may partially offset these positive effects. Nevertheless, we should keep in mind that seedling emergence represents only the initial phase of population recruitment; subsequent seedling survival under multifactorial stress may ultimately determine population persistence and expansion (Chambers et al. 1990; Schütz 2002).

### 4.2 Seedling Growth

The establishment success of newly emerged plant seedlings is determined by complex interactions among multiple ecological factors (Chambers et al. 1990). Our findings reveal that warmed temperatures generally promote seedling growth across most cushion plant species, as indicated by significant increases in shoot elongation and leaf production (Table 2; Table S1). This accelerated early growth provides alpine plants with critical ecological advantages: it facilitates rapid resource accumulation necessary to endure unpredictable extreme weather events during the brief growing season and enhances overwintering survival (Luscher et al. 2001; Chen, Chen, et al. 2024). Given the exceptionally constrained growing season characteristic of alpine ecosystems (Körner 2021), such rapid developmental progression is particularly vital, as failure to complete establishment before winter onset likely constitutes a major bottleneck for future population recruitment. In contrast to the positive effects of warming, we found that enhanced UV‐B radiation consistently exerted negative impacts on seedling growth parameters across most studied species (Table 2D,E; Figures 3 and 4). This observation suggests that although alpine cushion plants represent highly specialized adaptations to extreme alpine environments with naturally high UV‐B exposure (Aubert et al. 2014; Körner 2021), their early life stages remain susceptible to UV‐B‐induced physiological stress. These results corroborate an extensive body of research documenting the inhibitory effects of elevated UV‐B on plant growth and development (Bornman and Teramura 1993; Teramura 1983; Liang et al. 2006; Wang et al. 2008; Jansen et al. 2022). Of particular concern, stratospheric ozone depletion has already led to measurable increases in UV (including UV‐B) radiation across the Qinghai‐Tibet Plateau (Zhou et al. 1995, 2006; Chen et al. 2013). Our study highlights a critical climate change paradox: while warming may facilitate cushion plant recruitment through temperature‐mediated growth enhancement, the concomitant rise in UV‐B radiation could substantially mitigate these benefits. This antagonistic interaction between competing environmental drivers emphasizes the urgent need for comprehensive field studies to elucidate how these counteracting forces will ultimately determine the population dynamics and distribution patterns of alpine cushion plants under future climate scenarios.

### 4.3 Seedling Mortality

Our study uncovers a critical ecological constraint: both elevated temperatures and UV‐B radiation significantly accelerate seedling mortality rates in most cushion species (Figure 5; Figure S2). As cold‐adapted organisms with highly specialized adaptations, cushion plants display particular sensitivity to climate warming (Cranston et al. 2015; Chen, Chen, et al. 2024; Rai et al. 2024; Jandova et al. 2025). This thermal sensitivity is evidenced by the observed temperature‐driven mortality, aligning with previous reports of warming‐induced recruitment failure in species such as *A. oreophila* (Zhou et al. 2025) and *A. polytrichoides* (Chen, Chen, et al. 2024). Notably, this vulnerability is not limited to seedlings; mature cushion plants similarly exhibit increased mortality under warming conditions (Chen, Chen, et al. 2024). The added threat posed by UV‐B radiation further complicates the picture. While the precise mechanisms underlying these synergistic mortality effects require further investigation, they likely involve multiple pathways such as photosynthetic inhibition, DNA damage accumulation, phytohormone disruption, altered secondary metabolite profiles, and stomatal regulation impairment (Caldwell et al. 1995; Sharma et al. 2017; Jansen et al. 2022). These results imply that ongoing climate change may present a dual influence: while warming could initially have some positive effects on certain growth parameters, the combined effects of elevated temperatures and UV‐B radiation are likely to create increasingly unfavorable conditions for seedling establishment.

## 5 Summary and Perspectives

Previous studies have shown that the early recruitment stages (including seed germination and subsequent seedling growth) of cushion species such as *A. polytrichoides* and *A. oreophila* are influenced by a multitude of ecological factors, including temperature, moisture, and light availability, soil conditions, extreme climatic events, interspecific competition, and potential allelopathy from adjacent vegetation (Chen et al. 2020; Chen, Chen, et al. 2024; Chen, Qian, et al. 2024; Zhou et al. 2025). The current study provides supplementary evidence that while elevated temperatures may facilitate the seedling emergence and growth of various alpine cushion plants, they concurrently expedite the mortality process, leading to substantial seedling loss. Additionally, our findings reveal that seedling emergence can be severely restricted when seeds become lodged in deep soil layers, potentially due to light limitations that hinder seed dormancy breakage. The soil surface in alpine subnival ecosystems, characterized by gravel of varying sizes with substantial interstitial spaces, facilitates seed sinkage into deeper soil layers, imposing significant constraints on the germination process of numerous cushion species and further impeding their population recruitment and expansion. This underscores the need for careful consideration of seed burial depth in conservation and restoration efforts aimed at preserving and enhancing alpine cushion plant populations. For instance, in species whose seedling emergence is strongly limited by deep burial, surface sowing may be an effective strategy to improve population recruitment. More crucially, we observed detrimental effects of intense UV‐B radiation on the early recruitment stages of multiple cushion plants, spanning seedling emergence, growth, and survival. Taking together with previous evidence (Chen et al. 2020; Chen, Chen, et al. 2024; Chen, Qian, et al. 2024; Zhou et al. 2025), it is evident that the maintenance and recruitment of diverse cushion species in alpine ecosystems face considerable risks amidst ongoing climate change. Furthermore, our findings underscore species‐specific responses to environmental factors in cushion plant seedlings, which are important for understanding their growth dynamics and establishment. These differences may be attributed to the inherent biological attributes of the seeds—such as size, morphology, and physiological traits—which are closely linked to their respective life‐history strategies. However, several precautions must be taken before accurately predicting the development of alpine plant communities in tandem with the population dynamics of various cushion species. Firstly, as demonstrated in this study, different cushion species exhibit heterogeneous responses to environmental factors. Given that different cushion species exhibit distinct life‐history strategies, occupy diverse habitats (personal observation), and experience varying degrees of population suppression stress (Zhou et al. 2025), future research should prioritize uncovering the species‐specific driving mechanisms, processes, and potential trajectories shaping their population dynamics. Secondly, given that current conclusions are largely based on laboratory simulations (but see Chen, Chen, et al. 2024 for field studies), extensive field experiments in their native habitats are imperative for future studies to validate laboratory findings. In particular, we should focus on elucidating the coupling effects of multiple environmental factors—such as topographic variation, habitat heterogeneity, microclimate variability, and snow cover duration—on the population dynamics of cushion plants in natural community settings. Such insights are essential for comprehensively predicting how alpine cushion plant populations will respond to climate change. Thirdly, considering cushion plants' role as keystone species crucial to numerous ecological processes and functions, future investigations must give particular attention to the potential ecological ramifications of the population dynamics of multiple cushion species.

## Author Contributions

**Meihong Huang:** data curation (lead), formal analysis (supporting), investigation (lead), methodology (supporting), visualization (supporting), writing – original draft (supporting), writing – review and editing (supporting). **Pengfei Yang:** data curation (supporting), formal analysis (supporting), investigation (equal), methodology (supporting), visualization (equal), writing – original draft (supporting), writing – review and editing (supporting). **Jianying Xiang:** supervision (supporting), writing – review and editing (supporting). **Mengqiu Niu:** data curation (supporting), investigation (supporting), writing – review and editing (supporting). **Jie Lin:** data curation (supporting), investigation (supporting), writing – review and editing (supporting). **Jianguo Chen:** conceptualization (lead), data curation (supporting), formal analysis (lead), funding acquisition (lead), methodology (supporting), project administration (lead), resources (lead), supervision (lead), validation (supporting), visualization (supporting), writing – original draft (lead), writing – review and editing (lead).

## Conflicts of Interest

The authors declare no conflicts of interest.

## Supporting information


**Data S1:** ece372814‐sup‐0001‐supinfo.7z.
**Fig. S1:** Time required for seedling emergence initiation (cyan‐green) and for reaching maximum emergence percentage (light coral) across different cushion plant species under varying temperature (x‐axis) and UV‐B conditions (triangles: with UV‐B; circles: without UV‐B).Asterisks denote significant differences between UV‐B treatments at the same temperature (*P < 0.05; **P < 0.01; ***P < 0.001).
**Fig. S2:** Final seedling mortality under indicated experimental temperature and UV‐B conditions. No seedling emergence occurred for Saxifraga species at the lowest temperature with UV‐B treatment (see Fig. 2j, k)

## Data Availability

The datasets and R code generated during this study are provided as Supporting Information S1.
